# The effect of caregiver support interventions for informal caregivers of community-dwelling frail elderly: a systematic review

**DOI:** 10.5334/ijic.845

**Published:** 2012-08-10

**Authors:** Maja Lopez-Hartmann, Johan Wens, Veronique Verhoeven, Roy Remmen

**Affiliations:** Department of Primary and Interdisciplinary Care, University of Antwerp, Universiteitsplein 1, BE-2610 Wilrijk (Antwerp), Belgium; Department of Primary and Interdisciplinary Care, University of Antwerp Universiteitsplein 1, BE-2610 Wilrijk (Antwerp), Belgium, E-mail: johan.wens@ua.ac.be; Department of Primary and Interdisciplinary Care, University of Antwerp, Universiteitsplein 1, BE-2610 Wilrijk (Antwerp), Belgium, E-mail: veronique.verhoeven@ua.ac.be; Department of Primary and Interdisciplinary Care, University of Antwerp, Universiteitsplein 1, BE-2610 Wilrijk (Antwerp), Belgium, E-mail: roy.remmen@ua.ac.be

**Keywords:** frail elderly, caregivers, health services needs, demand

## Abstract

**Introduction:**

Informal caregivers are important resources for community-dwelling frail elderly. But caring can be challenging. To be able to provide long-term care to the elderly, informal caregivers need to be supported as well. The aim of this study is to review the current best evidence on the effectiveness of different types of support services targeting informal caregivers of community-dwelling frail elderly.

**Methods:**

A systematic literature search was performed in Medline, PsychINFO, Ovid Nursing Database, Cinahl, Embase, Cochrane Central Register of Controlled Trials and British Nursing Index in september 2010.

**Results:**

Overall, the effect of caregiver support interventions is small and also inconsistent between studies. Respite care can be helpful in reducing depression, burden and anger. Interventions at the individual caregivers’ level can be beneficial in reducing or stabilizing depression, burden, stress and role strain. Group support has a positive effect on caregivers’ coping ability, knowledge, social support and reducing depression. Technology-based interventions can reduce caregiver burden, depression, anxiety and stress and improve the caregiver’s coping ability.

**Conclusion:**

Integrated support packages where the content of the package is tailored to the individual caregivers’ physical, psychological and social needs should be preferred when supporting informal caregivers of frail elderly. It requires an intense collaboration and coordination between all parties involved.

## Introduction

The main challenge in primary health care is the ageing population and the accompanying multimorbidity, long-term care demands and costs. In the industrialized world, 25% of 65–69 year olds and 50% of 80–84 year olds are affected simultaneously by two or more chronic health conditions and need long-term care [1, 2]. It is estimated that the share of people over 80 years old will rise from 4% in 2010 to nearly 10% in 2050 [2]. Long-term care spending will rise accordingly. Across all OECD countries, long-term care costs now account for 1.5% of the gross domestic product (GDP) on average [2].

The frail elderly are either being cared for at home by formal and informal caregivers, or in nursing homes [3]. In order to be able to stay at home, elderly in need of long-term care require a range of services, health care as well as social services. Despite the fact that around 70% of long-term care users receive services at home, institutional care costs account for 62% of total spending in long-term care [2]. Governments are acknowledging this and are promoting initiatives that aim at maintaining the frail elderly at home longer and delaying nursing home admission. Innovative and integrated services to maintain the frail elderly at home for as long as possible need to be implemented.

The effectiveness of interventions to maintain independent living in elderly people has been profoundly studied in a systematic review and meta-analysis by Beswick et al. (2008). They showed that complex interventions can help elderly people to continue living at home [4]. Hallberg and Kristensson (2004) performed a review on case management interventions for community-dwelling frail older people [5]. Strikingly they identified only a few studies focusing on a family-oriented approach, including support for informal caregivers.

Informal caregivers are important resources for community-dwelling frail elderly. However, caring can be challenging, causing physical and mental health problems [6], financial problems and social isolation [7]. Caregiver depression, stress or burnout, among others, increase the risk of institutionalization of the person being cared for [8]. In order to provide long-term care to the frail elderly, their informal caregivers need to be supported as well. Cost-effective caregiver support policies can reduce the demand for expensive institutional care [2].

Systematic reviews on support for informal caregivers already exist, but they are targeted at specific groups of caregivers according to the patient’s chronic condition, for example, dementia, cancer, palliative care [9] or one specific type of support like group support or respite care [10, 11].

We do not want to limit our review to a single type of support service and its effects, a specific subgroup of caregivers or a single type of study design. Clinicians, in particular general practitioners in primary care tend to work with a broad range of caregivers and patients irrespective of their diagnosis. Every care giving situation is different and most caregiver’s needs cannot be answered by providing a single service. Therefore the aim of this study is to broadly review the current best evidence on different types of support services targeting informal caregivers of community-dwelling frail elderly.

Our research question is formulated using the PICO method [12]. ***What are the known effects of different types of support services targeting informal caregivers of community-dwelling frail elderly?***

The population (P) studied is the informal caregivers of community-dwelling frail elderly. For this study we define an **informal caregiver** as a person who provides care to a relative, friend or neighbor in need of long-term care on a regular basis, not through a professional or volunteer organization. There has to be a personal relationship between the caregiver and the care recipient. The **community-dwelling frail elder** in this study is a vulnerable older person still living at home but dependent on others for one or more Activities of Daily Life (ADL) on a long-term basis. The frail older person’s impairment is not linked to specific conditions.

As intervention (I) to be studied we are interested in a broad range of possible support services targeting informal caregivers. Studies comparing (C) different forms of support as well as studies comparing a form of support to usual care are eligible for inclusion.

We do not focus on a single caregiver-related outcome (O). We want to give an overview of the different outcome measures used in the included studies.

## Methods

### Search strategy and eligibility criteria

The methodology outlined in the Prisma Statement [13] was used as a guide for this systematic review. A literature search in Medline, PsychINFO, Ovid Nursing Database, Cinahl, Embase, Cochrane Central Register of Controlled Trials and British Nursing Index was carried out in September 2010. The search was limited to reviews and additional original effectiveness studies published in English, French, German or Dutch. A combination of indexing (Mesh) terms and free-text keywords concerning informal caregivers, frail elderly, caregiver needs and support interventions was used to find relevant articles. A detailed overview of the electronic search strategies used in the different databases is presented in Table 1. The multiple database search provided a total of 912 titles. After removing duplicates, 696 unique titles were stored in an EndNote X3 database.

### Study selection procedure

**Figure 1:**
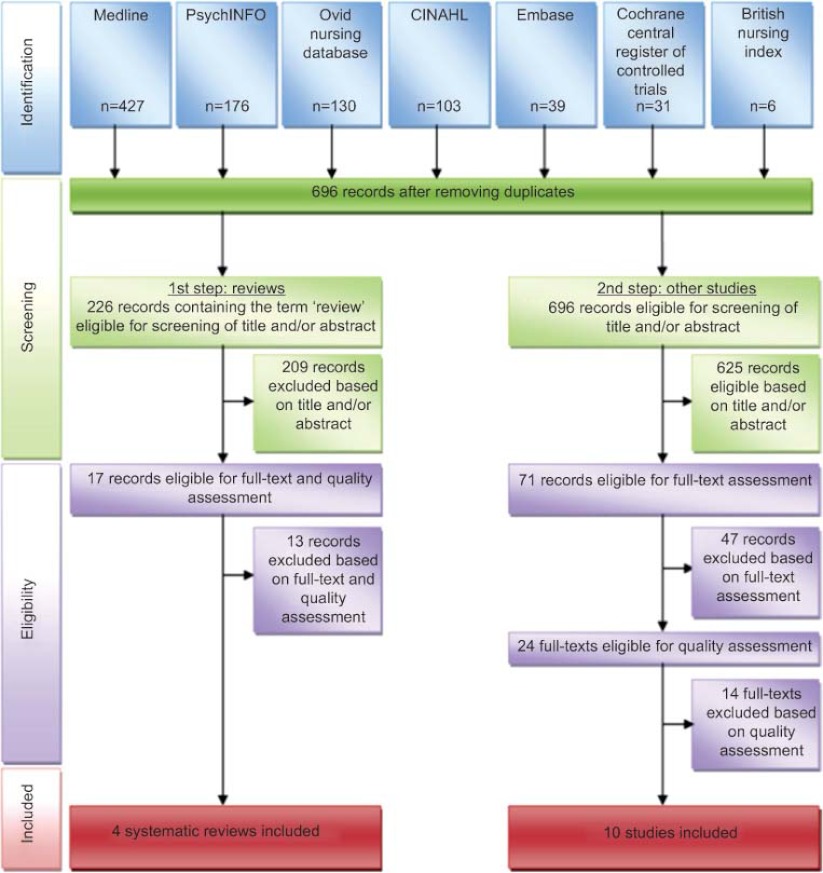
Study selection flow diagram (Prisma).

The selection procedure is presented in a flow diagram in Figure 1.

#### Step 1: review of reviews

Initially we only focused on the reviews. From the 696 unique references in our Endnote X3 database, 226 references contained the word review in any field. These references were screened on title and abstract by two researchers (MLH and JW). Reviews were included if they described community-based support services. The primary subject of the review had to be the informal caregiver and the informal caregiver had to care for a community-dwelling frail elder. Reviews about studies conducted in developing countries were excluded because of the difference in availability of formal support services. Most of the articles did not have the caregiver as the primary subject of the study, therefore they were excluded. After selection, 17 review articles remained to be assessed for methodological quality.

#### Step 2: review of primary studies

In a second step we went back to the set of 696 references to find additional primary studies that were not yet included in the selected review articles. All 696 articles were screened on title and abstract by two researchers (MLH and VV). This resulted in 71 articles that were eligible for assessment of the full text. After verifying that the articles met our inclusion criteria, 24 articles remained to be assessed on methodological quality.

### Quality assessment

The methodological quality of the studies was assessed using the Scottish Intercollegiate Guidelines Network’s methodology checklists [14]. Each study was assessed independently by two researchers (MLH and JW or RR or VV). Assessments were compared and discussed until mutual agreement. Only the articles that scored 10 out of 15 or more on quality were included in our literature review.

After consensus, four review articles were included [10, 11, 15, 16] and 13 review articles were excluded. Five of the excluded articles were actually not reviews, one was a duplicate, one was out of scope and six reviews did not meet our baseline quality score of 10 out of 15.

After quality appraisal of the additional primary studies, 10 articles were included in this review [17–26]. Three of these included articles [24–26] report on the same study but describe different outcomes (short-term, long-term and costs). Fourteen additional articles were excluded because of low quality scores.

## Results

This literature review will provide an overview of the relevant literature on the effects of different types of caregiver support. Results from four systematic reviews [10, 11, 15, 16] and 10 additional primary articles [17–26] will be discussed. Characteristics of the included studies are listed in Tables 2A and 2B.

### Outcomes

The number of different outcome variables used in each study varies from one [21] to 12 [23] (Table 2). Caregiver burden and depression were measured the most. Burden was assessed using three different instruments: the Zarit Burden Index (by Zarit ea, 1980) [19, 21, 22], the Montgomery-Borgatta Burden Scale (by Montgomery & Borgatta, 1986) [23–25] and the Preparedness for Caregiving Scale (by Archbold ea, 1990) [17, 20]. Depression was assessed with six different scales or subscales: The Center for Epidemiologic Studies—Depression Scale (by Radloff, 1977) [20, 22], The General Health Questionnaire (by Goldberg & Hillier, 1979) [24, 25], the Beck Depression Inventory (by Beck ea, 1967) [23], the Geriatric Depression Scale (by Yesavage ea, 1983) [23], the Generalized Contentment Scale (by Hudson, 1982) [19] and the Health and Daily Living Form (Billings ea, 1983) [18].

Only the outcome variables that were used in at least two different studies are being discussed, namely: depression, burden, stress, role strain, anger, anxiety, quality of life, coping ability, knowledge of resources, social support and economic burden.

### Types of support

Three main types of support are mentioned in the included studies: respite, psychosocial support and information and communication technology (ICT) support. Psychosocial support is studied at the individual caregiver’s level as well as at group level.

The four reviews cover separately: respite services [10, 11], psychosocial interventions (individual and group interventions) [15] and ICT support services [15, 16]. The 10 primary studies report on psychosocial support interventions providing education, information, coordination, counselling, psychological and emotional support, either in group [19, 22–26] or at the individual caregiver level [17, 18, 20, 21].

The findings on these three main types of support will be discussed in the following paragraphs and will be summarized in Table 3.

#### Respite

Respite services provide the caregiver with a temporary break in his care giving activities to improve the well-being of the caregiver. Two included systematic reviews report the effect of respite care on different caregiver outcomes.

Shaw et al. (2009) studied the effect of respite care on depression, burden, anger, anxiety and quality of life (Table 3) [10]. Pooled results show a positive effect of respite on caregiver burden after 2–3 month’s follow-up (Effect size (ES) -0.46; 95% Confidence interval (CI) -0.82 to -0.10) and after six months’ follow-up (ES -0.58; CI -1.06 to -0.11). Respite care had a positive impact on caregivers’ anger towards the care recipient (ES -0.38; CI -0.60 to -0.17). However, quality of life was significantly worsened after 6 to 12 months in caregivers receiving respite care (ES -0.22; CI -0.27 to -0.17). Although not statistically significant after pooling results, respite services tended to have a positive effect on depression and a negative effect on anxiety.

A systematic review and meta-analysis performed by Mason et al. (2007) studied the effect of respite care on caregivers’ depression, burden, quality of life and economic burden (Table 3) [11]. Mason et al. found a statistically significant positive effect of respite care on reducing depression (ES -0.32; CI -0.62 to -0.02) (Table 3). Respite care tended to have a positive effect on decreasing caregiver burden and a negative effect on improving quality of life although not significant. Economic evidence suggests that respite is at least as costly as usual care.

#### Psychosocial support

##### At the individual caregivers’ level

Contrary to respite services, where caregivers are provided a temporary break from caring, psychosocial support interventions aim at improving the caregivers’ ability to manage the caregiving situation. These services offer packages including education, skill-building, counselling, information and emotional support. The support is mostly given in the caregivers home. Cassie et al. (2008) reviewed studies evaluating individual support for caregivers [15]. They found that interventions at the individual caregivers’ level decrease caregiver depression (Table 3). They also improve the caregivers’ coping ability.

A randomized controlled trial (RCT) performed in the Netherlands by Melis et al. (2009) tested the effect of a problem-based home visiting programme for frail elderly on caregiver burden (Table 3) [21]. After 3 and 6 months, the treatment group did not show a significant decrease in burden compared to the control group. When analysing subgroups, caregivers sharing a household with the care recipient may have benefited, while the intervention might have had a negative effect on caregivers not living together with the frail older adult.

A quasi-experimental study by Horton-Deutsch et al. (2002) tested the effect of a multi component intervention for family caregivers [20]. No significant differences were found between treatment and control group for depression and global role strain (Table 3). The study found an important difference between the 2 nurses who provided the intervention. After eight weeks, caregivers in the treatment group of nurse A spent less hours on care giving because their patients improved. The nurse was able to assist as well the caregiver as the patient. In the treatment group of nurse B, the patients deteriorated and the caregivers spent more hours on care giving.

Another RCT evaluating the effect of an advanced nursing practice intervention (Dellasega et al. 2002) found that the intervention had a positive impact on caregivers’ outcomes (Table 3) [18]. Caregivers in the treatment group had significantly fewer depressive symptoms after 2 weeks (p≤0.05) and still after 4 weeks (p≤0.05) Additionally, they had significantly lower stress scores after 48 hours (p≤0.05). Working caregivers also had fewer disability days and less financial loss.

While the content of the support intervention in the previous studies could vary according to the caregivers’ needs, other individual interventions offer more defined educational and practical support like education about implementing a toilet regimen. Colling et al. (2003) performed a quasi-experimental study evaluating the effect of a continence program [17]. The study showed a significant decline in the caregivers perceived burden (Table 3).

##### Group interventions

In addition to the characteristics of interventions at the individual caregivers’ level, group interventions also have a social dimension. The interaction between group members can have an effect on caregivers that is impossible to achieve with individual support.

According to the review performed by Cassie et al. (2008) group interventions decrease depression and anxiety, increase their knowledge of community resources and increase their social support (Table 3) [15].

Toseland et al. (1992) performed an RCT to evaluate the effect of a group program for spouses of frail elderly veterans [23]. During 8 weeks spouses received weekly 2 hour group sessions. After the intervention no effect was found on depression. Significant decreases in subjective burden (p=0.009), and stress (p=0.031) were found (Table 3). Also significant increases in the use of active behavioural coping strategies (p=0.013), personal changes in the ability to cope with the caregiving situation (p<0.001) and knowledge of community resources (p=0.002) were found.

Three articles (Toseland et al. 2001, 2004, 2006) report separately on the short-term effects (2001), long-term effects (2004) and cost evaluation (2006) of an RCT evaluating a Health Education Group Program (HEP) for caregivers [24–26]. The program is a multicomponent, psychoeducational intervention program delivered in a structured group format. Compared to the control group, short-term benefits for the caregivers in the experimental group were found in reducing depression (p≤0.05). No effect was found on burden and role strain. The intervention increased coping ability (p≤0.01), knowledge of community services (p≤0.01) and social integration (p≤0.05) (Table 3). After one year the intervention was still effective in reducing depression (p≤0.05), increasing coping ability and knowledge of community services (p≤0.01) (Table 3). Still no positive effects were found on burden and role strain. The results of the cost-effectiveness study indicate that total costs and outpatient costs were significantly lower in the intervention group compared to the control group (Table 3).

Smith and Toseland (2006) adapted the design of the HEP to create a telephone support program for caregivers [22]. Results show that the intervention had a strong positive effect on the adult child caregivers, but no effect on the spouse caregivers. Adult child caregivers had a greater reduction in depressive symptoms (p≤0.05), stress of pressing problems (p≤0.05), role strain (p≤0.05) and personal strain (p≤0.001). They felt more effective in coping with pressing problems (p≤0.05). There was also a significant increase in knowledge of community services (p≤0.001) and in social support (p≤0.01) (Table 3).

A quasi-experimental study by Demers and Lavoie (1996) showed contradictory results (Table 3) [19]. The intervention had a stabilizing effect on the level of depressive symptoms in the treatment group (p<0.05) but they experienced an unexpected increase of subjective burden (p<0.05), while caregivers’ burden in the control group decreased.

##### Information and communication technology

More recent literature focuses on the effect of information and communication technology to support caregivers. Cassie et al. (2008) reviewed the use of telephone and computer services to provide support and education to caregivers at home [15]. They found that technology-based interventions could reduce depression, burden and anxiety (Table 3). Magnusson et al. (2004) conclude that information and communication technology interventions could reduce caregiver stress and promote optimal coping (Table 3) [16].

## Discussion

### Evidence

This systematic overview identifies different types of interventions to support informal caregivers of community-dwelling frail elderly. The evidence is summarized in Table 3. Some evidence exists for the effectiveness of respite care, interventions at individual caregiver level, group support and information and communication technology. Overall, the effect of caregiver support interventions is small and also inconsistent between studies.

Respite care can be helpful in reducing depression, burden and anger. Anxiety and quality of life do not seem to improve when offering respite services.

Interventions at the individual caregiver level can be beneficial in reducing or stabilizing depression, burden, stress and role strain. Surprisingly few studies evaluating individual interventions measure the caregivers’ coping ability and knowledge.

Group support has proven to have a positive effect on caregivers’ coping ability and knowledge as well as on social support. Studies evaluating group support find a positive effect of the intervention on caregivers’ depression. The effect of group support on caregivers’ burden is not consistent. Some studies find a positive effect, while others find no or negative effects. It is possible that participating in the group sessions causes burden instead of unburden the caregiver, while it may entail that the caregiver for example has to find sitting services for the elder during the group sessions.

Technology-based interventions can reduce caregiver burden, depression, anxiety and stress and improve the caregiver’s coping ability.

No single intervention can answer all relevant physical, psychological and social needs of an informal caregiver caring for a frail elderly at home.

### Integrated services

The term integration is often used differently in literature [27]. One can look at integration from a patients’ as well as from a care provision perspective.

In a holistic patient-centered approach, support services should integrate all relevant physical, psychological and social needs of the patient. But needs from patients can differ from their informal caregivers’ own needs. Support services targeting the needs of frail elderly are not necessarily concurrently beneficial for their informal caregivers. Therefore, integrated support services should pay special attention to supporting the caregivers specific physical, psychological and social needs as well.

On the other hand, integration can also mean a collaboration between different professionals, within and between the cure and care sector, or within and between primary, secondary and tertiary care setting. Informal caregivers are important resources for frail elderly, but their contribution in the care as a care provider is often taken for granted [28]. Informal caregivers are often sandwiched between being a care provider and a person in need of care. It is important that this ambiguous position is acknowledged by professional care providers. Today, this is often not yet the case.

A well-supported informal caregiver is an essential partner in the long-term care for the frail elderly, since no professional care system will ever be able to cover all of the elder’s needs [28]. Support for the informal caregiver should be integrated in all services aiming at delaying institutionalization of the frail elderly. In the future more research should be done on integrated services for the elderly that explicitly incorporate support for the informal caregiver.

### Weaknesses

Using the search term *Frail Elderly* might not have captured all relevant articles concerning this population. Frail elderly as a concept is new in research literature. In Pubmed the Mesh-term Frail Elderly was introduced in 1991. Gobbens et al. (2010) reviewed the literature to identify the different definitions used to describe frail elderly and proposed a new conceptual definition of frailty [29].

In this study we reviewed the literature on the effectiveness of support services. The fact that we focused on quantitative data is a weakness. In addition to evidence of effectiveness, evidence of feasibility, appropriateness and meaningfulness found in qualitative studies could have told us a lot about how an intervention is related to the context in which it is given and how the intervention is experienced by the population.

The variety of outcome variables and measures used in the studies made it difficult to adequately compare results. When designing an evaluation study it is important to carefully select the most adequate outcome measures to assess interventions. Melis et al. (2009) only assessed the effect on caregiver burden and time spent on care [21]. No significant differences were found between study groups for these outcomes. However, concluding that the intervention did not benefit the caregivers is too premature. While the intervention mainly focused on advice and coordination of care, other outcome measures like coping ability or knowledge would also have been interesting to assess. Future research should pay special attention to matching the aim and content of the intervention to the most adequate outcome measures.

### Strengths

We identified evidence for the effectiveness of caregiver support interventions irrespective of the elderly’s disease entity. Caregiver needs are highly individual and can change over time. They are related to more aspects than only the elderly’s health status. A profound assessment is essential to identify caregiver needs, priorities, cultural aspects and existing resources. Such an assessment will help clinicians to work out the most appropriate support strategy together with the caregiver. Often a combination of different types of services is necessary to answer the actual needs of the individual caregiver.

### Recommendations for future research

More research is needed to explore the concept of optimal caregiver support. Who is best placed to perform a needs assessment and coordinate integrated caregiver support? Is it the role of the general practitioner or are in fact other professionals better placed? Caregivers exist all over the world, but their support needs can be different because of cultural habits and the healthcare system of the country they live in. In further research special attention should go to the influence of the caregivers’ characteristics and context on the outcome.

Concerning the design of future studies, RCT’s might not be the most adequate method to evaluate the effectiveness of caregiver support interventions. Other (mixed-)methods including economic evaluations and qualitative methods should be considered. At present, few studies did incorporate long-term effect evaluation. Future research should focus on the effect of integrated services over a longer period of time.

## Conclusion

The heterogeneity in aim, content and intensity of the studied interventions demonstrates that defining ‘best caregiver support’ is not easy if not impossible.

While respite care is aimed at unburdening the caregiver by temporarily taking over the care for the elderly, psychosocial and educational support aims at strengthening the caregiver in his ability to better manage and cope with the care giving role.

Integrated support packages where the content of the package is tailored to the individual caregivers’ physical, psychological and social needs should be preferred when supporting informal caregivers of frail elderly. It requires an intense collaboration and coordination between all parties involved.

Although this literature review does not have a direct link with integrated care, we are convinced that informal caregivers of community-dwelling frail elderly can benefit from integrated support services. Additionally, informal caregivers play an important role in the delivery of integrated care to the frail elderly. This paper may not add a lot of new insights to integrated care, however, the fact that this paper focuses on the informal caregiver in the first place instead of the patient is not common in existing research.

These findings are important for future programme development. In Belgium for instance, the central Government induced bottom up approach for new and innovative projects with a common purpose to keep frail elderly in their homes, including support for informal caregivers [30]. To inform responsible stakeholders, evidence should be compiled and readily available. We hope that our contribution will support stakeholders when designing new avenues for the support of informal caregivers of community-dwelling frail elderly.

## Competing interests

The authors declare that they have no competing interests.

## Authors’ contributions

MLH carried out the literature search, study selection, quality appraisal, data analysis and drafted the manuscript. JW, VV and RR participated in the study selection, quality appraisal and drafting the manuscript. All authors read and approved the final manuscript.

## Acknowledgements

We thank Cil Leytens who helped us in finding full texts of articles.

## Reviewers

**Joel Ankri**, MD, PhD, Professor, Head of the “Centre of Gerontology”, Ste Perine Hospital (APHP); Head of “Health, Environment, Aging” Research Unit (EA2506), University of Versailles, Paris, France.

**Yves Couturier**, M.s.s., Ph.D, Chaire de recherche du Canada sur les pratiques professionnelles d'intégration de services en gérontologie, Centre de recherche sur le vieillissement, Centre de santé et des services sociaux, Institut universitaire de gériatrie de Sherbrooke, Québec, Canada.

**Debby L. Gerritsen**, PhD, senior researcher Department of Primary and Community Care, Center for Family Medicine, Geriatric Care and Public health, Radboud University Nijmegen Medical Centre, the Netherlands.

## Figures and Tables

**Table 1:**
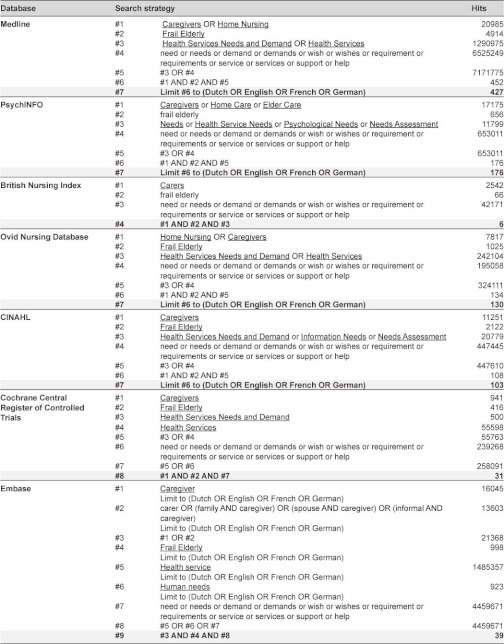
Search strategy.

Mesh terms are underlined.

**Table 2A:**
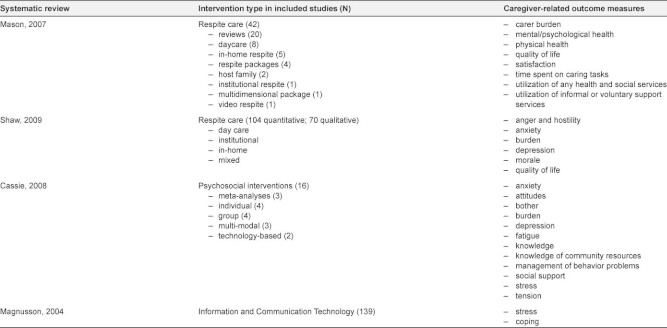
Characteristics of included reviews.

**Table 2B:**
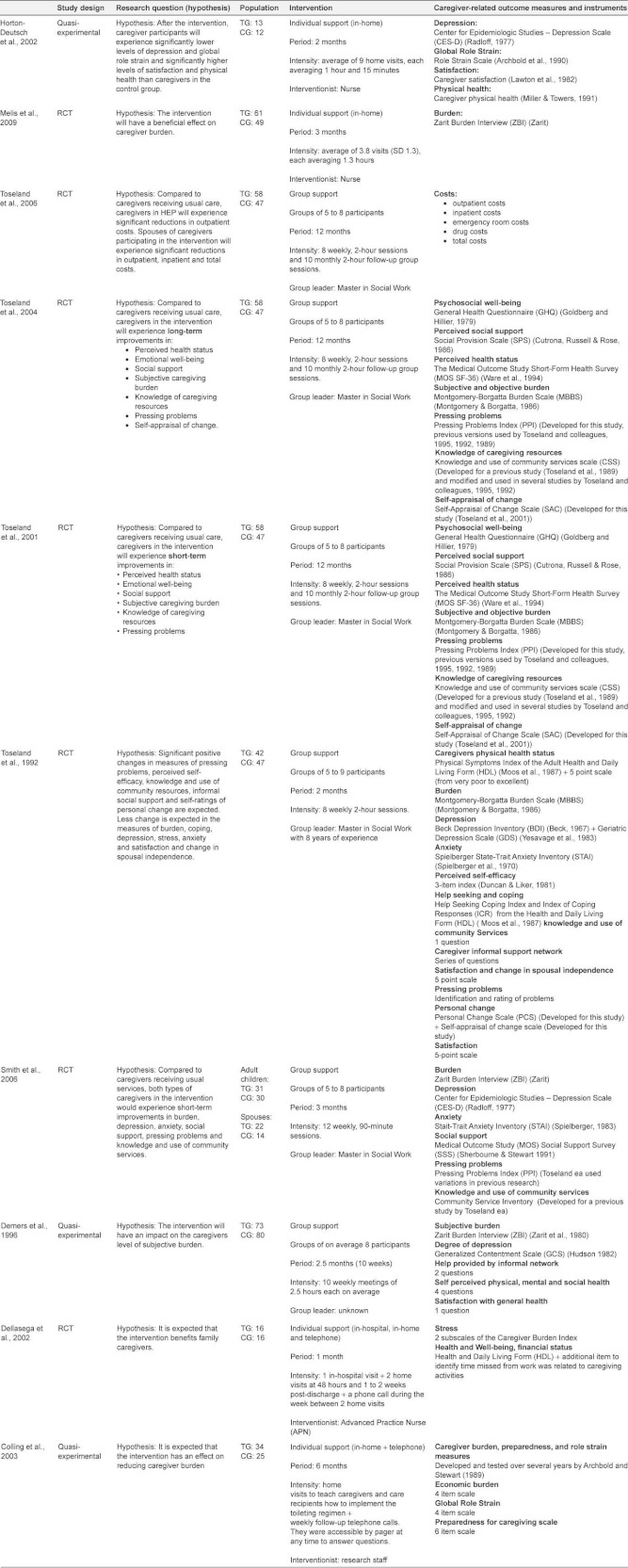
Characteristics of included primary studies.

TG=test group; CG=control group.

**Table 3:**
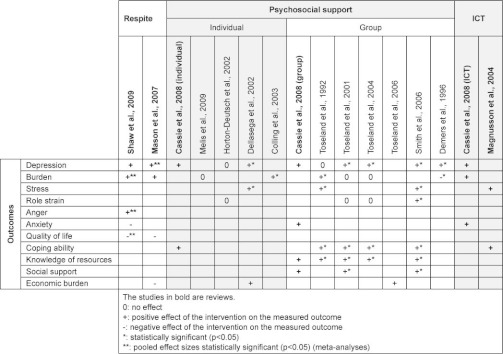
Results from systematic reviews and primary studies.
